# Seeking and accessing professional support for child anxiety in a community sample

**DOI:** 10.1007/s00787-019-01388-4

**Published:** 2019-08-13

**Authors:** Tessa Reardon, Kate Harvey, Cathy Creswell

**Affiliations:** 1 School of Psychology and Clinical Language Sciences, University of Reading, Reading, UK; 2 Departments of Experimental Psychology and Psychiatry, University of Oxford, Oxford, UK

**Keywords:** Help-seeking, Barriers, Parents, Children, Anxiety disorders

## Abstract

There is a lack of current data on help-seeking, and barriers to accessing professional support for child anxiety disorders. This study aimed to provide current data on the frequency and type of parental help-seeking, professional support received, and parent-reported barriers/facilitators in the context of child anxiety, and to explore factors associated with help-seeking, and parent-reported barriers among help-seekers and non help-seekers. We conducted a survey of help-seeking in parents of 222 children (aged 7–11) with elevated anxiety symptoms identified through screening in schools, 138 children of whom met diagnostic criteria for an anxiety disorder. Almost two-thirds (64.5%) of parents of children with an anxiety disorder reported seeking help from a professional; in 38.4% of cases parents reported that their child had received support from a professional to help manage and overcome their anxiety difficulties, and < 3% had received evidence-based treatment (CBT). Frequently reported parental barriers related to difficulties differentiating between developmentally appropriate and clinically significant anxiety, a lack of help-seeking knowledge, perceived negative consequences of help-seeking, and limited service provision. Non-help seekers were more likely than help seekers to report barriers related to thinking a child’s anxiety may improve without professional support, and the absence of professional recognition. Findings identify the need for (i) tools for parents and primary school staff to help identify children who may benefit from professional support to overcome difficulties with anxiety; and (ii) increased evidence-based provision for child anxiety disorders, including delivery within schools and direct support for parents.

**Electronic supplementary material:**

The online version of this article (10.1007/s00787-019-01388-4) contains supplementary material, which is available to authorized users.

## Introduction

Anxiety disorders are the most prevalent mental health disorders experienced across the lifespan, with an average age of onset of 11 years [1]. Childhood anxiety disorders have a significant negative impact on educational, social, and health functioning, and are associated with continued anxiety and other mental health disorders in adulthood [2], and substantial economic burden [3]. Cognitive behavioural therapy (CBT) is the most well-evaluated treatment for anxiety disorders in children [4], with evidence for long-term positive outcomes [5, 6]. However, poor rates of access to treatment for mental health problems in children are widely reported [7–9]. Studies report lower rates of treatment access among children with anxiety disorders compared to children with behavioural disorders [8, 10, 11], although there is a lack of current UK data on the number of families who seek and access professional support for child anxiety disorders, and we know little about the type of support families receive.

The unmet need in relation to child mental health has prompted calls to improve access to effective early intervention [12]. Children typically rely on a parent or caregiver to seek help on their behalf so it is important that interventions designed to improve access to professional support for child mental health problems address barriers parents face throughout the help-seeking process [13]. Studies that explore factors associated with use of child mental health services help illustrate who does and who does not access treatment, and help identify potential barriers to accessing treatment. For example, if parents perceive a child’s difficulties to be problematic, and perceive a negative impact on family life, and need for professional help, a child is more likely to access mental health services than if parents do not recognise a child’s difficulties, associated impact or need for help [14]. Parents’ own experience of mental health problems and use of mental health services, and a child’s symptom severity are also associated with child mental health service use [15, 16]. However, few studies have explored help-seeking, specifically in the context of child anxiety disorders; given that reported rates of treatment access are lower for anxiety disorders compared to behavioural disorders, there may be barriers to help-seeking that are unique to anxiety difficulties. To identify key areas to target to improve treatment access, it is also pertinent to establish parents’ own views on barriers/facilitators associated with seeking and accessing professional support in the context of child anxiety. Salloum, et al. [17] described parental perceptions of barriers to treatment access within a sample of parents of children who received treatment for anxiety, but given that so few families reach services, we also need to consider the experiences of parents who have not sought and/or accessed support for their child. Indeed, qualitative interviews we conducted with parents of children with anxiety disorders identified in the community illustrate the range of difficulties parents can face recognising a child’s anxiety difficulties, identifying the need for support, contacting professionals for help or advice, and receiving support from professionals [18]. It is therefore important to establish barriers to both seeking help and obtaining support to help manage and overcome a child’s anxiety difficulties. Establishing the extent to which parents report different types of barriers, and improving understanding of who experiences which types of barriers would help inform targeted interventions to both promote help-seeking and ensure more families receive appropriate support.

### Aims

This study aimed to build on the qualitative findings to date [18] by providing quantitative data on help-seeking within a community sample of parents of children with elevated anxiety symptoms, and within a subsample where the child met diagnostic criteria for an anxiety disorder. Specifically, we aimed to provide current data on the frequency and type of (i) parental help-seeking, and (ii) professional support families receive to help manage and overcome a child’s anxiety difficulties. We also aimed to (iii) provide quantitative data on the frequency and type of parental reported barriers and facilitators to seeking and accessing professional support for their child’s anxiety. Additionally, we set out to explore (iv) the child and parent characteristics associated with seeking professional help and parent-reported barriers, and (v) differences in parent-reported barriers among those who have and those who have not sought professional help.

## Methods

### Recruitment procedure

Participants were recruited through screening in primary/junior schools in England. To ensure the sample included participants from different geographic regions and captured a varied demographic profile, we identified a random sample of primary/junior schools in England to approach, stratified by the Department of Education’s 10 geographic regions and the number of children on the roll eligible for free school meals. Between May 2016 and May 2017 538 schools were invited to participate in the study, and 62 were recruited. Recruited schools were from across all 10 geographic regions (North–West, North–East, East Midlands, Yorkshire and the Humber, East Midlands, West Midlands, London (outer and inner), East of England, South East, and South West), with 22–731 pupils on the school roll, and a mean of 15.88% (SD 11.51) children eligible for free school meals. Schools that were invited but did not participate usually did not respond to the invitation (476 schools; > 90%), and where schools declined the invitation, the most frequently cited reason was a lack of available time. Invited schools that did not take part did not differ from recruited schools in terms of number of pupils on the school roll or mean number of children eligible for free school meals.

Recruited schools distributed study information and anxiety screening questionnaires (Spence Children’s Anxiety Scale-Parent Version; SCAS-P) to all parents/caregivers of children in year groups 3–6 (aged 7–11 years). Parents/caregivers (hereby referred to as parents) provided informed consent and completed the SCAS-P on paper or online. Researchers then visited the school to administer corresponding questionnaires with the children whose parents had provided consent, and their class teachers. In total, parents of 10,338 children were invited to take part, 1884 provided consent, and 1881 parents, 1617 children and 1579 teachers completed screening questionnaires. In cases where screening questionnaire scores indicated the child had elevated anxiety symptoms,^1^ the parent was invited to take part in a follow-up diagnostic assessment (Anxiety Disorder Interview Schedule-Parent Version; ADIS-P) and to complete follow-up questionnaire measures relating to their child’s anxiety (Child Anxiety Impact Scale-Parent Version; CAIS-P), their views and experiences surrounding seeking professional help for their child’s anxiety and their own mental health (Depression Anxiety Stress Scales; DASS-21) and mental health service use. Diagnostic assessments were administered by telephone, and follow-up questionnaires were posted to parents and returned in a pre-paid envelope. We invited parents of 639 children with elevated anxiety symptoms to take part in the follow-up, parents of 255 children agreed to take part, and parents of 222 children both completed the diagnostic assessment and returned the help-seeking questionnaire. Child anxiety symptoms (SCAS-P) were significantly higher among parents who took part in the follow-up, than those who were invited but did not participate (*t* [634] = 3.74, *p* < 0.001).

### Participants

The sample included parents of 222 children aged 7–11 years with elevated anxiety symptoms, 138 children of whom met DSM-5 criteria for at least one anxiety disorder. Participant characteristics for the total sample and the subsample are provided in Table 1. Across the total sample the majority of parents who participated were female (*n* = 206, 92.8%), with a mean age of 40.46 years (SD 5.92), and 99 (44.6%) completed higher education. Children had a mean age of 9.63 (SD 1.22), and 107 (48.2%) were female. Among children with an anxiety disorder, 58 (42.0%) children had 2 or more anxiety diagnoses, and the most common anxiety disorders were Generalised Anxiety Disorder (*n* = 87, 63.0%), Social Anxiety Disorder (*n* = 52, 37.7%), Specific Phobia (*n* = 34, 24.6%) and Separation Anxiety Disorder (*n* = 31, 22.5%). Non-anxiety comorbid disorders were Obsessive Compulsive Disorder (*n* = 8, 5.8%), Attention Deficit and/or Hyperactivity Disorder (ADHD; *n* = 17, 12.3%), and Oppositional Defiant Disorder (ODD; *n* = 2, 1.4%); an additional 6 children who did not meet criteria for an anxiety disorder had behavioural disorders (ADHD, *n* = 4; ODD, *n* = 2).

**Table 1:** Sample characteristics

	Anxiety disorder sample (*n* = 138)	Total sample (*n* = 222)
Child gender		
Female, *n* (%)	63 (45.7)	107 (48.2)
Child age		
Mean (SD)	9.79 (1.18)	9.63 (1.22)
Parent gender		
Female, *n* (%)	133 (96.4)	206 (92.8)
Parent age		
Mean (SD)	40.38 (5.49)	40.46 (5.92)
Parent ethnicity		
White British, *n* (%)	128 (92.8)	200 (91.2)
Family SES		
Higher/professional, *n* (%)	64 (46.4)	105 (47.3)
Other employed, *n* (%)	66 (47.8)	102 (45.9)
Unemployed, *n* (%)	5 (3.6)	9 (4.1)
Parent education		
School completion, *n* (%)	20 (14.5)	35 (15.8)
Further education, *n* (%)	62 (44.9)	85 (38.3)
Higher education/postgraduate, *n* (%)	53 (38.4)	99 (44.6)
SCAS-P (total score)		
Mean (SD)	39.37 (15.72)	33.99 (15.73)
SCAS-C-27 (total score)		
Mean (SD)	29.36 (14.73)	28.33 (14.31)
SCAS-T-20 (total score)		
Mean (SD)	12.04 (8.65)	11.94 (8.04)
CAIS-P		
Total score, mean (SD)	26.26 (13.53)	21.46 (14.22)
School, mean (SD)	11.86 (6.78)	10.06 (7.09)
Social, mean (SD)	7.95 (5.53)	6.36 (5.54)
Home/family, mean (SD)	6.44 (4.41)	5.05 (4.32)
Parent-rated need for support for child’s anxiety		
Child benefit from professional support		
Mean (SD)	3.00 (1.16)	2.73 (1.24)
*n* (%) somewhat/strongly agree	102 (73.9)	142 (64.0)
Parent benefit from support to help child		
Mean (SD)	3.01 (1.12)	2.78 (1.20)
*n* (%) somewhat/strongly agree	106 (76.8)	151 (68.3)
DASS-21		
Total score, mean (SD)	15.60 (12.36)	13.90 (11.69)
Anxiety, mean (SD)	3.32 (3.77)	2.77 (3.49)
Stress, mean (SD)	7.85 (5.34)	7.13 (5.19)
Depression, mean (SD)	4.42 (4.80)	4.00 (4.54)
Parent contact with professional about mental health problems	94 (68.1)	147 (66.2)
GP, *n* (%)	86 (62.3)	127 (58.0)
Therapist/counsellor, *n* (%)	63 (45.7)	94 (42.3)
Psychiatrist, *n* (%)	11 (8.0)	18 (8.2)
Parent-rated professional support not helpful *n* (%)	10 (7.2)	12 (5.4)
Parent-rated professional support very helpful/ extremely helpful *n* (%)	40 (29.0)	73 (32.9)
Primary anxiety diagnosis		
Separation anxiety disorder, *n* (%)	14 (10.2)	
Social anxiety disorder, *n* (%)	32 (23.4)	
Generalised anxiety disorder, *n* (%)	67 (48.9)	
Specific phobia, *n* (%)	15 (10.9)	
Selective mutism, *n* (%)	1 (0.7)	
Other specified AD, *n* (%)	8 (5.8)	
Primary anxiety disorder, CSR		
Mean (SD)	4.86 (0.82)	
Presence anxiety diagnosis		
Separation anxiety disorder, *n* (%)	31 (22.5)	
Social anxiety disorder, *n* (%)	52 (37.7)	
Generalised anxiety disorder, *n* (%)	87 (63.0)	
Specific phobia, *n* (%)	34 (24.6)	
Selective mutism, *n* (%)	1 (0.7)	
Other specified AD, *n* (%)	9 (6.5)	
Presence of other diagnoses		
OCD, *n* (%)	8 (5.8)	
ADHD, *n* (%)	17 (12.3)	21 (9.5)
ODD, *n* (%)	2 (1.4)	4 (0.9)

*Other Specified AD *other specified anxiety disorder, *CSR *clinical severity rating, *OCD *obsessive compulsive disorder, *ADHD *attention deficit and/or hyperactivity disorder, *ODD *oppositional defiant disorder

### Measures

#### Spence Children’s Anxiety Scale—parent-, child-, and teacher-report versions (SCAS-P; SCAS-C-27; SCAS-T-20)

The Spence Children’s Anxiety Scale is a well-evaluated 38-item questionnaire measure designed to assess symptoms of DSM child anxiety disorders, with parent- and child-reported versions [19–24]. To reduce administration time, the obsessive compulsive behaviours and physical injury fears subscales were not included in the child-reported questionnaire, on the basis of the DSM5 classification of anxiety disorders and item functioning analyses [21]. A corresponding teacher-reported version of the SCAS that consists of 20 items derived from the SCAS-C/P (SCAS-T-20) [21] was also used.

#### Anxiety disorder interview schedule: parent version (ADIS-P)

The ADIS-P is a reliable and valid structured parent interview designed to assess a child’s diagnostic status, including an assessment of DSM anxiety diagnoses and common comorbid disorders [25–27]. Minor amendments were made to enable diagnoses consistent with DSM5 following personal communication with the authors. As per the guidelines, diagnoses and Clinical Severity Ratings (CSRs) 4–8 were assigned where the child met the diagnostic criteria, and the disorder with the highest CSR was assigned as the primary disorder. Assessments were administered by graduate and undergraduate psychology students, and for each assessor the first 20 assessments were discussed with an experienced diagnostician and consensus reached. After a minimum of 20 assessments, and once assessors obtained a minimum kappa/ICC of 0.85, then one in six subsequent interviews were discussed. Inter-rater reliability within the assessment team was excellent (diagnoses, kappa = 0.91, CSR ICC = 0.95).

#### Child anxiety impact scale: parent version (CAIS-P)

The CAIS-P is a 27-item parent-reported questionnaire measure of the impact of a child’s anxiety symptoms on school, social and home/family activities, with good psychometric properties [28, 29]. Two items considered inappropriate for pre-adolescent children (going on a date, having a boyfriend/girlfriend) were omitted.

#### Help-seeking views and experiences questionnaire

This questionnaire measure was developed for this study and provided information on the following:

(i) *Parental help-seeking and professional support received for their child’s anxiety difficulties.* These questions were based on questions used in the *National survey of mental health in children and young people in Great Britain* [7]*.* Parents were asked (i) if they had ever contacted a professional for help or advice about their child’s difficulties with anxiety, and if so to indicate the professional/s they contacted; and (ii) if their child had ever received support from a professional to help manage or overcome their child’s difficulties with anxiety, and if so to indicate the type of support received and who provided the support. To assess informal help-seeking, parents were asked to indicate other people they had spoken to for help or advice about (i) their child’s anxiety, and (ii) getting support from a professional to help with their child’s anxiety.

(ii)* Parental perceived need for professional support for their child’s anxiety.* Parents were asked to rate the extent to which they felt (i) their child may benefit from professional support, and (ii) the parent may benefit from support from a professional to help their child manage/overcome their difficulties with anxiety.

(iii) *Parent-perceived barriers and facilitators to seeking and accessing professional support for their child’s anxiety difficulties*. Parents rated the extent to which 44 items stopped/made it harder for them to seek/access professional support. Items were rated on a 4-point Likert scale (*not stopped/not made it harder* = 0 to *very much stopped/made it very much harder* = 3). In cases where the parent reported contacting a professional for help or advice, parents also rated the extent to which 30 items encouraged/made it easier for them to seek/access professional support for their child’s difficulties with anxiety. Facilitator items were also rated on a 4-point scale (*not encouraged/not made it easier* = 0 to *very much encourage/made it very much easier* = 3). Barrier and facilitator items were developed based on findings from a systematic review of parental perceived barriers/facilitators to child mental health treatment [13] and qualitative interviews with parents of children with anxiety disorders [18]; and refined and finalised following consultation and piloting with members of a local research advisors group comprising of parents of children who have experienced mental health difficulties. Responses to all barrier/facilitator items were summed to produce total scores. Barriers and facilitators relate to four distinct stages in the help-seeking process identified in underpinning qualitative work (recognising a child’s anxiety difficulties; recognising the need for professional support; contacting professionals; receiving support) [18], and items related to each stage were summed to produce four respective barrier/facilitator subscale scores.

#### Depression Anxiety and Stress Scales (DASS-21)

The DASS-21 [30, 31] is a brief self-report questionnaire that was used to measure parents’ symptoms of anxiety, depression and stress.

#### Parent mental health service use

Parents were asked if they had spoken to a professional about their own mental health using an item sourced from similar surveys [15, 32]. Where applicable, parents also indicated the professional they had spoken to and rated the helpfulness of the support they received (*not at all helpful* = 0 to *extremely helpful* = 4).

### Data analytic approach

#### Descriptive statistics

Parental help-seeking, support received, and parent-reported barriers were examined within the total sample, and within the subsample where the child met criteria for an anxiety disorder. The number and proportion of parents who reported seeking professional help for their child’s anxiety difficulties were calculated, together with the number/proportion who reported seeking help from school staff, General Practitioners (GPs), mental health specialists, and other non-professionals. Similarly, the number and proportion of parents who reported that their child received professional support were calculated, together with the type of support (CBT, counselling, parenting support, recommended strategies/resources) and professional who provided the support [teacher/school staff, GP, National Health Service (NHS) mental health specialist, private professional, other professional]. In relation to parent-reported barriers, the number/proportion who endorsed each barrier (and 95% CI), mean item score (and 95% CI) for each barrier, total barriers score, and four barrier subscale scores were examined. Among parents who reported seeking professional help for their child’s anxiety, corresponding descriptive statistics were examined for parent-reported facilitators.

#### Parental help-seeking

Characteristics associated with parental help-seeking were then examined. Bivariate analyses (independent *t *test, *X*^2^ test) were used to explore whether the following child/parent characteristics were associated with seeking professional help or not: demographic variables (child’s age, child’s gender, parent education, parent occupation), child’s anxiety symptoms/associated impairment (SCAS-P and CAIS-P), parent-perceived need for professional support for their child, parental mental health (DASS-21), and parental use of mental health services/rated helpfulness of mental health services. A logistic regression model was then used to examine the unique contribution of individual parent/child characteristics in identifying whether parents had sought professional help for the child’s anxiety or not across the total sample. Parent/child characteristics that were significantly associated with seeking professional help in the bivariate analyses, together with presence/absence of a child anxiety disorder were entered into the logistic regression model using a block-enter method.

#### Parent-reported barriers

Bivariate analyses (Pearson’s *r*, *X*^2^ test) were also used to explore child/parent characteristics associated with parent-reported barriers (total barrier scores and barrier subscale scores), and independent *t* tests were used to examine differences in parent-reported barriers between parents who had sought professional support and those who had not. A logistic regression model was used to further explore whether particular barriers were uniquely associated with seeking/not seeking help (i.e., to determine whether some barriers deter parents from contacting professionals, and others become relevant following contact with professionals). To explore particularly pertinent barriers, for each subscale the first and second rank-ordered barriers (based on the proportion of parents who endorsed the item) were used as predictor variables, and whether the parent had sought help/not sought help as the outcome variable. Barrier scores for the first and second rank ordered barriers for each subscale were entered into the logistic regression model using a block-enter method to identify the unique contribution of individual barriers in identifying whether parents had sought help or not. To control for the presence/absence of an anxiety diagnosis and demographic variables that were significantly associated with parent-reported barriers in bivariate analyses, these variables were also included in the model. VIF and tolerance statistics were examined for evidence of multicollinearity in regression models.^2^

## Results

### Frequency and type of help-seeking and support received

Parent-reported help-seeking and support received to help with their child’s anxiety difficulties are displayed in Table 2. Almost two-thirds (64.5%) of parents of children with an anxiety disorder reported contacting a professional for help or advice, and just over half (52.7%) of parents in the total sample reported contacting a professional. The majority of parents who sought help contacted school staff (58.7% of parents of a child with an anxiety disorder; 47.3% in the total sample), and a smaller proportion contacted a GP (37.7%/27.7%, respectively), someone specialising in mental health (27.5%/21.0%, respectively) or another professional (13.0%/9.9%, respectively). Interestingly, almost all parents (> 90%) across the whole sample reported some informal help-seeking and had spoken to someone for help or advice about their child’s anxiety. Almost three-quarters (73.5%) of parents of children with anxiety disorders, and 60.4% across the total sample, also reported seeking informal advice specifically about getting professional support for their child.

**Table 2:** Frequency and type of help-seeking and support accessed

	Anxiety disorder sample (*n* = 138)	Total sample (*n* = 222)
Contacted a professional for help or advice, *n* (%)	89 (64.5)	117 (52.7)
Professional contacted		
GP/family doctor, *n* (%)	52 (37.7)	62 (27.9)
Teacher or support staff, *n* (%)	81 (58.7)	105 (47.3)
Someone specialising in child mental health, *n* (%)	38 (27.5)	48 (21.0)
School nurse, *n* (%)	1 (1.2)	5 (2.3)
Telephone helpline, *n* (%)	3 (2.2)	4 (1.8)
Other health/social care professional, *n* (%)	18 (13.0)	22 (9.9)
Informal help-seeking about child’s anxiety, *n* (%)	136 (98.6)	210 (94.6)
Spoken to/used		
Friends, *n* (%)	124 (89.9)	176 (79.3)
Family, *n* (%)	127 (92.0)	199 (89.6)
Other parents, *n* (%)	109 (79.0)	152 (68.5)
Work colleagues, *n* (%)	69 (50.0)	96 (43.2)
Internet, *n* (%)	99 (71.7)	134 (60.6)
Online parent forums, *n* (%)	26 (18.8)	36 (16.5)
Books, *n* (%)	44 (31.9)	64 (29.2)
Informal help-seeking about seeking professional support for child, *n* (%)	101 (73.2)	134 (60.4)
Friends, *n* (%)	60 (43.5)	79 (35.6)
Family, *n* (%)	62 (44.9)	84 (37.8)
Other parents, *n* (%)	41 (29.7)	56 (25.7)
Work colleagues, *n* (%)	36 (26.1)	46 (21.4)
Internet, *n* (%)	62 (44.9)	83 (37.4)
Online parent forums, *n* (%)	14 (10.1)	20 (9.0)
Received help from a professional, *n* (%)	53 (38.4)	72 (32.4)
Type of help received		
CBT with child and/or parent, *n* (%)	3 (2.2)	5 (2.3)
Counselling for child, *n* (%)	20 (14.5)	27 (12.2)
Parenting support, *n* (%)	12 (8.7)	15 (6.8)
Professional recommended resources/strategies/books, *n* (%)	21 (15.2)	29 (13.1)
Other type of help, *n* (%)	21 (15.2)	27 (12.2)
Unspecified school support, *n* (%)	5 (3.6)	8 (3.6)
Nurture/emotional group at school, *n* (%)	3 (2.2)	5 (2.3)
Speech therapy, *n* (%)		1 (0.5)
Art/play/drama/alternative therapy, *n* (%)	6 (3.5)	
Medication, *n* (%)	2 (1.4)	
Unspecified support, *n* (%)	4 (2.9)	
Who provided help		
GP/family doctor, *n* (%)	5 (3.6)	8 (3.6)
Teacher or school staff, *n* (%)	30 (21.7)	42 (18.9)
NHS professional specialising in child mental health, *n* (%)	21 (15.2)	29 (13.1)
Private professional, *n* (%)	6 (4.3)	7 (3.2)
Charity, *n* (%)	9 (6.5)	10 (4.5)
School nurse, *n* (%)	1 (0.7)	2 (0.9)
School counsellor, *n* (%)	2 (1.4)	2 (0.9)
Paediatrician, *n* (%)	2 (1.4)	3 (1.4)
Occupational/speech therapist, *n* (%)	1 (0.7)	2 (0.9)

In relation to receiving professional support, 38.4% of parents of children with an anxiety disorder (and 32.4% across the total sample) reported that their child had received some type of support from a professional to help manage or overcome anxiety difficulties. Families received a range of types of professional support, including child counselling (14.5% of children with anxiety disorders; 12.2% across the total sample), professionals recommending resources/strategies (15.2%/13.1%, respectively), and parenting support (8.7%/6.8%, respectively). Notably, only 2.3% of parents across the total sample reported that their child had received CBT. Parents most frequently reported receiving professional support from school staff (21.7% of parents of children with an anxiety disorder, 18.9% across the total sample), followed by NHS mental health specialists (15.2%/13.1%, respectively).

### Parent-reported barriers

Parent-reported barriers across the total sample, and among those where the child met criteria for an anxiety disorder are displayed in Table 3. Mean total barrier scores were 36.95 (SD 25.15) in the total sample, and 43.39 (SD 24.93) among parents of children with anxiety disorders.

**Table 3:** Parent-reported barriers to seeking and accessing professional support for child anxiety difficulties

	Anxiety disorder sample (*n* = 138)		Total sample (*n* = 222)	
	Mean (SD) 95% CI	*n* (%) endorsed 95% CI	Mean (SD) 95% CI	*n* (%) endorsed 95% CI
***Recognising child’s anxiety difficulty barriers (total)***	***8.33 (5.17) 7.45–9.23***		***7.41 (5.21) 6.71–8.12***	
My child has always been anxious	1.10 (1.06) 0.92–1.28	81 (58.7) 50.0–67.0	0.87 (1.00) 0.73–1.00	111 (50.0) 43.2–56.8
My child’s anxiety comes and goes in phases^a^	1.55 (1.01) 1.36–1.74	101 (73.2)^a^ 65.0–80.4	1.38 (1.08) 1.23–1.52	153 (68.9)^a^ 62.4–74.9
I’m not sure if my child’s anxiety is normal^a^	1.38 (1.10) 1.19–1.57	96 (69.6)^a^ 61.2–77.1	1.20 (1.08) 1.05–1.34	141 (63.5)a 56.8–69.9
I think my child’s anxiety is normal	0.78 (0.97) 0.61–0.95	62 (44.9) 36.5–53.6	0.82 (1.01) 0.68–0.95	101 (45.5) 38.8–52.3
I’m not sure if my child has anxiety difficulties or other difficulties	1.35 (1.14) 1.15–1.55	89 (64.5) 55.9–72.4	1.23 (1.51) 1.07–1.39	134 (60.4) 53.6–66.8
My child has other difficulties that are more serious than anxiety	0.73 (1.01) 0.56–0.90	55 (39.9) 31.6–48.5	0.63 (0.97) 0.50–0.76	77 (34.7) 28.4–41.3
My child doesn’t understand that she/he has anxiety difficulties	0.98 (1.09) 0.79–1.16	73 (52.9) 44.2–61.4	0.82 (1.04) 0.68–0.96	104 (46.9) 40.1–53.6
I don’t know other people who have had anxiety difficulties	0.49 (0.86) 0.34–0.64	40 (29.0) 21.6–37.3	0.49 (0.87) 0.37–0.61	65 (29.3) 23.4–35.7
***Recognising the need for professional support barriers (total)***	***12.47 (8.35) 11.03–13.90***		***10.88 (8.17) 9.79–11.98***	
Teachers or other professionals have never suggested my child would benefit from professional help^b^	1.25 (1.21) 1.05–1.46	81 (58.9)^b^ 50.0–67.0	1.11 (1.18) 0.95–1.27	120 (54.1)^b^ 47.3–60.3
Talking to my child about her/his anxiety may make the problem worse	1.01 (1.04) 0.83–1.19	74 (53.6) 44.9–62.1	0.88 (1.01) 0.74–1.01	110 (49.6) 42.8–56.3
I don’t want my child to think she/he has a problem^b^	1.36 (1.10) 1.18–1.55	95 (68.8)^b^ 60.4–76.4	1.22 (1.10) 1.07–1.37	141 (63.5)^b^ 56.8–69.9
Family life is busy or we have lots of other things going on in the family	0.89 (1.08) 0.70–1.07	64 (46.4) 37.9–55.1	0.82 (1.03) 0.68–0.96	100 (45.1) 38.4–51.8
Professionals can’t help with anxiety difficulties in children	0.34 (0.71) 0.22–0.46	30 (21.7) 15.2–29.6	0.28 (0.65) 0.20–0.37	42 (18.9) 14.0–24.7
My child’s anxiety may improve without professional help	0.99 (1.05) 0.81–1.17	77 (55.8) 47.1–64.2	0.95 (1.02) 0.81–1.09	120 (54.1)b47.3–60.7
I want us to manage my child’s anxiety as a family	0.95 (1.05) 0.77–1.13	72 (52.2) 43.5–60.5	0.92 (1.03) 0.78–1.06	116 (52.3) 45.5–59.0
I feel a sense of failure or blame as a parent	1.15 (1.14) 0.96–1.35	78 (56.5) 47.8–64.9	0.98 (1.11) 0.83–1.13	111 (50.0) 43.2–56.8
People I know may blame me or judge me or think I am a bad parent	0.85 (1.10) 0.66–1.04	58 (42.0) 33.7–50.7	0.66 (0.99) 0.52–0.79	80 (36.0) 29.7–42.7
People see anxiety or mental health difficulties as a weakness	0.98 (1.14) 0.79–1.18	66 (47.8) 39.3–56.5	0.83 (1.07) 0.69–0.97	96 (43.2) 36.6–50.0
I don’t want my child to be labelled	1.08 (1.18) 0.88–1.28	73 (52.9) 44.2–61.4	0.98 (1.15) 0.82–1.13	109 (49.1) 42.3–55.9
I don’t want other people to know about my child’s difficulties	0.59 (0.91) 0.43–0.74	48 (34.8) 26.9–43.4	0.48 (0.83) 0.37–0.59	68 (30.6) 24.6–37.1
My child is worried about what other children will think about her/him getting help	1.05 (1.18) 0.84–1.25	69 (50.0) 41.4–58.6	0.86 (1.12) 0.71–1.01	95 (42.8) 36.2–49.6
***Contacting professionals barriers (total)***	***11.05 (7.80) 9.70–12.39***		***8.95 (7.66) 7.92–9.98***	
I don’t know who to ask for help^c^	1.32 (1.29) 1.12–1.51	89 (64.5)^c^ 55.9–72.9	1.12 (1.15) 0.96–1.27	122 (55.0)^c^ 48.2–61.6
I don’t know what help is available for children with anxiety difficulties^c^	1.53 (1.09) 1.34–1.71	100 (72.5)^c^ 64.2–79.7	1.36 (1.13) 1.21–1.51	150 (67.6)^c^ 61.0–73.6
I don’t trust information I’ve read online about professional help for anxiety difficulties in children	0.64 (0.88) 0.49–0.79	54 (39.1) 30.9–47.8	0.51 (0.81) 0.40–0.62	73 (32.9) 26.7–39.5
I don’t know if GPs can provide advice on anxiety difficulties	0.85 (1.07) 0.66–1.03	61 (44.2) 35.8–52.1	0.67 (0.99) 0.54–0.80	82 (36.9) 30.6–43.7
I don’t know if teachers can provide advice on anxiety difficulties	1.02 (1.05) 0.83–1.20	79 (57.3) 48.6–65.6	0.84 (1.02) 0.70–0.97	105 (47.3) 40.6–54.1
I am afraid if I talk to a professional it will raise concerns about my parenting	0.62 (0.90) 0.46–0.77	54 (39.1) 30.9–47.8	0.46 (0.81) 0.35–0.57	68 (30.6) 24.6–37.1
Professionals won’t listen to me or won’t take me seriously	0.86 (1.07) 0.68–1.05	63 (45.7) 37.2–54.3	0.66 (0.97) 0.53–0.79	83 (37.4) 31.0–44.1
Professionals will blame me	0.58 (0.95) 0.42–0.74	43 (31.2) 23.6–39.6	0.43 (0.83) 0.32–0.54	56 (25.2) 19.7–31.5
Teachers don’t know much about anxiety difficulties in children	1.01 (1.08) 0.83–1.20	76 (55.1) 46.4–63.5	0.81 (1.02) 0.68–0.95	102 (46.0) 39.3–52.7
My GP doesn’t know me and/or my child	1.03 (1.23) 0.82–1.24	63 (45.7) 37.2–54.3	0.84 (1.15) 0.69–0.99	88 (39.6) 33.2–46.4
GPs don’t know much about anxiety difficulties in children	0.76 (1.00) 0.58–0.93	57 (41.3) 33.0–50.0	0.61 (0.92) 0.49–0.73	79 (35.6) 29.3–42.3
It is difficult to make an appointment at my GP surgery	1.09 (1.18) 0.89–1.29	61 (44.2) 35.8–52.9	0.94 (1.12) 0.79–1.08	105 (47.3) 40.6–54.1
***Receiving professional support barriers (total)***	***12.24 (9.30) 10.62–13.84***		***10.15 (9.01) 8.92–11.37***	
Professionals have dismissed my concerns about my child in the past	1.01 (1.20) 0.80–1.21	64 (46.4) 37.9–55.1	0.78 (1.14) 0.63–0.93	79 (35.6) 29.3–42.3
Professionals don’t think my child needs professional help	0.64 (0.94) 0.47–0.80	50 (36.2) 28.2–44.8	0.53 (.88) 0.41–0.65	68 (30.6) 24.6–37.1
Professionals only offer parenting courses	0.59 (1.02) 0.41–0.77	38 (27.5) 20.3–35.8	0.46 (.92) 0.33–0.58	50 (22.5) 17.2–28.6
There isn’t any professional help available for children with anxiety difficulties	0.57 (0.91) 0.41–0.72	43 (31.2) 23.6–39.6	0.47 (.83) 0.35–0.58	62 (27.9) 22.1–34.3
It is a battle to access professional help	1.35 (1.24) 1.13–1.56	81 (58.7) 50.0–67.0	1.08 (1.20) 0.92–1.25	111 (50.0) 43.2–56.8
It is difficult to get a referral to a specialist service^d^	1.49 (1.23) 1.28–1.70	89 (64.5|) 55.9–72.4^d^	1.18 (1.21) 1.02–1.34	120 (54.1) ^d^ 47.3–60.7
There are long waiting times for specialist services^d^	1.57 (1.27) 1.35–1.79	89 (64.5) 55.9–72.4^d^	1.27 (1.27) 1.10–1.45	121 (54.5)^d^ 47.7–61.2
I can’t afford to pay for private professional help	1.44 (1.34) 1.21–1.67	79 (57.3) 48.6–65.6	1.27 (1.32) 1.09–1.45	116 (52.3) 45.5–59.0
My child’s behaviour is not disruptive at school	0.93 (1.17) 0.73–1.12	62 (44.9) 36.5–53.6	0.85 (1.14) 0.70–1.01	94 (42.3) 35.8–49.1
My child’s school does not prioritise mental health	0.69 (1.04) 0.51–0.87	50 (36.2) 28.2–44.8	0.58 (.95) 0.45–0.70	71 (32.0) 25.9–38.6
My child’s school has limited resources	0.92 (1.07) 0.74–1.10	68 (49.3) 40.7–57.9	0.80 (1.04) 0.66–0.94	97 (35.6) 37.1–50.5
***Total barriers***	***43.39 (24.93) 39.13–47.65***		***36.95 (25.15) 33.59–40.31***	

Bold italic values are the subscale totals

^a^First and second rank ordered *Recognising child’s anxiety difficulty* barriers based on % endorsed

^b^First and second rank ordered *Recognising the need for professional support* barriers based on % endorsed

^c^First and second rank ordered *Contacting professionals* barriers based on % endorsed

^d^First and second rank ordered *Receiving support* barriers based on % endorsed

#### Barriers to recognising a child’s anxiety difficulties

The most frequently endorsed barriers to recognising a child’s anxiety difficulties, were *my child’s anxiety comes and goes in phases* (73.2% of parents of children with an anxiety disorder; 68.9% across the total sample) and *I’m not sure if my child’s anxiety is normal* (69.6% of parents of children with an anxiety disorder; 63.5% across the total sample). Notably, less than a third (across both the total sample and those with a child with an anxiety disorder) endorsed *I don’t know other people who have had anxiety difficulties* as a barrier.

#### Barriers to recognising the need for professional support

The most commonly endorsed barrier to recognising the need for professional support was *I don’t want my child to think she/he has a problem* (68.8% of parents of children with anxiety disorders; 63.5% across the total sample). More than half of parents across the whole sample also rated *teachers or other professionals have never suggested my child would benefit from professional help*, *my child’s anxiety may improve without professional help*, *I want us to manage my child’s anxiety as a family*, and *I feel a sense of failure or blame as a parent* as barriers. Notably, only one-fifth of the sample endorsed *professionals can’t help with anxiety difficulties in children* as a barrier.

#### Barriers to contacting professionals

*I don’t know what help is available for children with anxiety difficulties* and *I don’t know who to ask for help* were the most frequently endorsed barriers to contacting professionals (72.5%/64.5%, respectively, among parents of children with anxiety disorders; and 67.6%/55.0%, respectively, across the total sample).

#### Barriers to receiving professional support

In relation to accessing or obtaining professional support to help manage and overcome a child’s difficulties, more than half of all parents rated *it is difficult to get a referral to a specialist service, there are long waiting times for specialist services, I can’t afford to pay for private professional help*, and *it is a battle to access professional help* as barriers; and almost two-thirds (64.5%) of parents with anxiety disorders endorsed the barriers related to referral difficulties and waiting times.

### Parent-reported facilitators

Table 4 displays reported facilitators among those parents who had contacted a professional for help or advice about their child’s anxiety. More than 80% of parents who had sought help rated *my child’s anxiety got worse*, *my child’s anxiety impacts on his/her life* and *I am desperate to get help for my child* as encouraging them or making it easier for them to seek professional help. The majority of help-seekers (> 80%) also endorsed the facilitators *I trust the teachers at my child*’*s school* and *Teachers at my child’s school are understanding and supportive*. In relation to receiving professional support, parents most frequently endorsed facilitators related to parental effort (*I have not given up asking for help*, 61.5%; *I have pushed hard to get professional help for my child*, 51.3%).

**Table 4:** Parent-reported facilitators among parents who reported seeking help (*n* = 117)

	Mean (SD) 95% CI	*n* (%) endorsed 95% CI
***Recognising child’s anxiety difficulty facilitators (total)***	***7.78 (3.80) 7.07–8.49***	
My child’s anxiety got worse^a^	2.18 (0.97) 2.00–2.36	104 (88.9)^a^ 81.7–93.9
My child’s anxiety difficulties are serious^a^	1.64 (1.05) 1.44–1.83	92 (78.6)^a^ 70.1–85.7
My friends/family think that my child has anxiety difficulties	1.58 (1.17) 1.36–1.80	84 (71.8) 62.7–79.7
Something happened to trigger or cause my child’s anxiety difficulties	1.20 (1.22) 0.98–1.43	65 (55.6) 46.1–64.7
A professional told me my child may have anxiety difficulties	1.17 (1.28) 0.93–1.41	58 (49.6) 40.2–59.0
***Recognising the need for professional support facilitators (total)***	***8.87 (5.02) 7.93–9.80***	
My friends/family think my child may benefit from professional help	1.54 (1.21) 1.31–1.77	79 (67.5) 58.2–75.9
My child wants professional help	1.01 (1.18) 0.79–1.23	55 (47.0) 37.7–56.5
I am desperate to get help for my child^b^	1.77 (1.13) 1.56–1.98	94 (80.3)^b^ 72.0–87.1
I cannot manage my child’s anxiety without professional help or support	1.38 (1.12) 1.17–1.58	81 (69.2) 60.0–77.4
My child’s anxiety impacts on his/her life^b^	2.25 (.88) 2.08–2.41	106 (90.6)^b^ 83.8–95.2
A professional advised me to seek help for my child	0.91 (1.18) 0.69–1.13)	50 (42.7) 36.6–52.6
***Contacting professionals facilitators (total)***	***13.27 (6.31) 12.09–14.44***	
I know how to talk to professionals	1.37 (1.15) 1.16–1.59	80 (68.4) 59.1–76.7
Professional listen to me	1.12 (1.02) 0.93–1.30	75 (64.1) 54.7–72.8
My GP is understanding and supportive	0.73 (0.99) 0.54–0.91	49 (41.9) 32.8–51.4
Teachers at my child’s school are understanding and supportive^c^	1.73 (1.06) 1.53–1.92	96 (82.1)^c^ 73.9–88.5
I trust my GP	1.04 (1.07) 0.85–1.24	67 (57.3) 47.8–66.4
I trust the teachers at my child’s school^c^	1.81 (1.04) 1.61–2.00	98 (83.8)^c^ 75.8–89.9
Teachers at my child’s school know my child	1.85 (1.11) 1.64–2.06	94 (80.3) 72.0–87.1
There is a clear point of contact at my child’s school	1.80 (1.23) 1.57–2.02	86 (73.5) 64.5–81.2
I have read online about the help that is available for children with anxiety difficulties	0.88 (1.14) 0.66–1.09	48 (41.0) 32.0–50.5
I know other parents who have spoken to professionals about their child’s anxiety or other mental health difficulties	0.91 (1.13) 0.70–1.12	53 (45.3) 36.1–34.8
***Receiving professional support facilitators (total)***	***7.97 (6.74) 6.71–9.23***	
I paid a private professional for an assessment or support for my child’s anxiety	0.35 (0.91) 0.17–0.52	12 (10.3) 5.4–17.2
I have pushed hard to get professional help for my child^d^	1.11 (1.22) 0.88–1.33	60 (51.3)^d^ 41.9–60.6
I have not given up asking for help^d^	1.40 (1.27) 1.17–1.64	72 (61.5)^d^ 52.1–70.4
I have contacted different professionals to try to get help for my child	1.01 (1.15) 0.79–1.22	58 (49.6) 40.2–59.0
My child meets the required criteria to access specialist services	0.80 (1.21) 0.57–1.03	39 (33.3) 24.9–42.6
My GP referred my child to a specialist service	0.73 (1.17) 0.51–0.94	36 (30.8) 22.6–40.0
My child’s school helped with a referral to a specialist service	1.00 (1.25) 0.77–1.23	50 (42.7) 33.6–53.2
My child has received professional help for another mental health or physical health difficulty	0.65 (1.12) 0.45–0.86	32 (27.4) 19.5–36.4
My child has received support through school for an academic/learning related difficulty	0.97 (1.21) 0.75–1.20	50 (42.7) 33.6–52.2
***Total facilitators***	***37.92 (18.01) 34.55–41.27***	

Bold italic values are the subscale totals

^a^First and second rank-ordered *Recognising child’s anxiety difficulty* facilitators based on % endorsed

^b^First and second rank-ordered *Recognising the need for professional support* facilitators based on % endorsed

^c^First and second rank-ordered *Contacting professionals* facilitators based on % endorsed

^d^First and second rank-ordered *Receiving support* facilitators based on % endorsed

### Factors associated with parental help-seeking

Findings from bivariate analyses and a logistic regression examining factors associated with parental help-seeking are reported in Online Resource 1 and 2, respectively. Parent-reported child anxiety symptoms (SCAS-P), impact of child anxiety (CAIS-P total) and impairment related to home/family activities (CAIS-home), were each significantly higher among help-seekers than non-help seekers in both the total sample and the anxiety disorder subsample. Teacher-reported child anxiety symptoms (SCAS-T-20), and parent-reported impairment related to school/social activities were also significantly higher among help-seekers than non-help seekers across the total sample. Across the total sample and the anxiety disorder subsample, parental perceived need for support for their child, both in relation to direct support for their child and support for the parent to enable them to help their child, and parent self-reported mental health symptoms were each significantly higher among help-seekers than non-help seekers. Significantly more help-seekers than non-help seekers also reported contact with a mental health specialist for their own mental health, among the total sample and the anxiety disorder subsample. No significant differences were found between help-seekers and non-help seekers on demographic variables or parent ratings on the helpfulness of the professional support they received for their own mental health difficulties.

Parental perceived need for professional support for their child (Odds Ratio 4.23 [95% CI 2.08–8.83]), parents’ own contact with a mental health specialist (Odds Ratio 2.63 [95% CI 1.33–5.19]), and impairment related to home/families activities (Odds Ratio 1.14 [95% CI 1.03–1.27]) were each uniquely associated with help-seeking for a child’s anxiety difficulties. Notably, after controlling for other variables, parent perceived need for support for themselves to enable them to help their child was significantly associated with *not* seeking help for their child’s anxiety difficulties (Odds Ratio 0.35 [95% CI 0.17–0.73]). Child anxiety symptoms (SCAS-P), presence of an anxiety diagnosis and parental mental health symptoms (DASS-21) were not uniquely associated with parental help-seeking.

### Factors associated with parent-reported barriers

As shown in Online Resource 3, the same variables were significantly associated with total barrier scores in the total sample and the anxiety disorder subsample. Total barrier scores were significantly higher among parents with lower educational qualifications; and significantly higher among parents who reported contact with a mental health specialist for their own mental health compared to those who had not had contact with a mental health specialist. Child anxiety symptoms (SCAS-P, SCAS-C-27), associated impairment (CAIS-P, CAIS-subscales), parental perceived need for support for their child, and parent mental health symptoms (DASS-21) were also each significantly correlated with total barrier scores.

### Parent-reported barriers associated with help-seeking

Differences between help-seekers and non-help seekers on total barrier and barrier subscale scores are displayed in Online Resource 1, and similar patterns were observed across the total sample and the anxiety disorder subsample. Total barrier scores were significantly higher among parents who had sought help for their child’s anxiety than those who had not sought help. Unsurprisingly, barriers related to contacting professionals and receiving support were also significantly higher among help-seekers than non-help seekers; however there were no significant differences between these two groups in reported barriers related to recognising a child’s anxiety difficulties and recognising the need for professional support.

As shown in Online Resource 4, individual barriers related to recognising a child’s anxiety difficulty did not make a unique contribution in identifying help-seekers/non-help seekers (*my child’s anxiety comes and goes in phases*, *I’m not sure if my child’s anxiety is normal*), and nor did barriers related to a lack of knowledge surrounding help-seeking (*I don’t know who to ask for help, I don’t know what help is available for children with anxiety difficulties*). Two barriers related to recognising the need for support however were negatively associated with seeking professional help (*my child’s anxiety may improve without professional help*, odds ratio, 0.60 [95% CI 0.40–0.89]; *teachers or other professionals have never suggested my child would benefit from professional help*, odds ratio, 0.57 [95% CI 0.40–0.82]). In contrast, the parent-reported barrier related to difficulty getting a referral to a specialist service was positively associated with seeking professional help (odds ratio, 2.36 [95% CI 1.28–4.35]). After controlling for these frequently endorsed barriers, the presence of an anxiety diagnosis was also associated with help-seeking (odds ratio 3.12 [95% CI 1.52–6.43]).

## Discussion

### Main findings

This study aimed to provide current and more detailed information on help-seeking for child anxiety difficulties than previously reported. Our findings illustrate a substantial unmet need in relation to child anxiety. We found that more than 60% of children with anxiety disorders had not received any professional support to help manage and overcome their difficulties with anxiety, and in about one third of cases parents had not sought help from a professional. Parents who had sought help, usually spoke to a member of school staff; and families who received professional support reported a range of support, but only a very small minority (< 3%) reported that their child had received evidence-based treatment (CBT). Indeed, while there is evidence that 50–60% of pre-adolescent children recover from anxiety disorders without treatment over a 2–3 year period [2, 33], even those children who recover without treatment are at an estimated 50% increased risk for poor functioning in adulthood [34].

Importantly, our findings show that factors other than a child’s level of need are the most important determinants of seeking help for child anxiety difficulties. Consistent with the broader literature surrounding child mental health service use [14], we found that parents who perceived that their child may benefit from professional support were more likely to have sought help than those who did not perceive a need for help, but interestingly, after controlling for other child and parent factors, parents who had *not* sought help were more likely to perceive that they themselves may benefit from professional support than those who had sought help for their child. One possible interpretation of this finding is that many parents may feel they would benefit from some support, but this is not enough to prompt actual help-seeking behaviour; or it may relate to a belief that seeking professional help for a child’s anxiety may facilitate access to direct support for a child rather than for a parent. In line with previous studies, our findings also indicate that parents’ own mental health plays a role in help-seeking for child anxiety [14, 15], but we found that it was parents’ prior use of mental health services, not their mental health symptoms, that was uniquely associated with help-seeking for a child. We did not find evidence that parents’ perceptions surrounding the helpfulness of their mental health support was associated with help-seeking for their child, but it is possible this is a reflection of the limited variability in perceived helpfulness reported in this sample.

We also set out to establish parents’ own views on barriers/facilitators to seeking and accessing support for child anxiety difficulties. Our findings support previous evidence that parental perceptions of a child’s mental health difficulties are an important determinant of parental help-seeking [14], and this study illustrates particular difficulties parents commonly face identifying anxiety problems in children. The majority of parents of children with elevated anxiety, including those where the child met diagnostic criteria, reported barriers related to differentiating between developmentally appropriate and clinically significant anxiety in children. This echoes previous qualitative findings [18] that indicate that uncertainty about whether a child’s anxiety is *normal* or not related to the fact that anxiety is a common childhood experience and can be readily attributed to a child’s temperament, and therefore presents a key, anxiety-specific help-seeking barrier. Our findings also highlight the key role primary school staff play in relation to parental help-seeking for child anxiety, with many parents citing a failure of teachers or other professionals to suggest a child needs help as a barrier, and the vast majority of help-seekers endorsing trustworthy and/or understanding teachers as facilitators. Parents also frequently experienced a range of other barriers that made them hesitant or reluctant to seek professional support. Interestingly, although negative views surrounding the effectiveness of mental health treatment are cited as a parent-reported barrier elsewhere [18], only a minority of parents in this study endorsed *professionals can’t help with anxiety difficulties in children* as a barrier. Rather, concerns surrounding negative consequences for the child, specifically: not wanting their child to think they have a problem; a belief the anxiety may improve without professional help; a desire to manage the difficulties as a family; and feeling a sense of blame as a parent, more frequently deterred parents from seeking professional support. A lack of help-seeking knowledge is reported elsewhere as a barrier to parental help-seeking [18], and we similarly found that many parents considered a lack of knowledge about who to ask for help and what help is available as barriers in the context of child anxiety. Parents commonly reported barriers related to limited service provision, including long waiting times and referral difficulties; and the level of parental effort required to get help was also apparent, with parents not giving up and pushing hard to get help the most frequently reported facilitators related to accessing support.

The findings also indicate that particular barriers deter or prevent parents from seeking help from professionals; and additional barriers tend to become relevant after parents contact professionals, and make it hard for families to access support. It is therefore not surprising that help-seekers reported more barriers than non-help seekers, and other variables associated with help-seeking were also associated with parent-reported barriers (child’s symptoms and impairment, parental perceived need for help, parental mental health symptoms and mental health service use); indicating that barriers accumulate as a parent progresses through the help-seeking process. In particular, we found that parents who had sought help were more likely to report barriers related to limited service provision than those who had not sought help. Some recognition barriers were common across help-seekers and non-help seekers, such as the transient nature of the child’s anxiety, and uncertainty about whether the anxiety is normal or not. However, parents who had not sought help were more likely to report the absence of professional recognition of the child’s need for help and a belief that a child’s anxiety may improve without professional help as barriers, suggesting these are particularly pertinent deterrents to help-seeking.

### Strengths and limitations

This study focused on parental help-seeking specifically in the context of anxiety in pre-adolescent children, and by doing so identified areas to target to promote help-seeking and access to support within this population where anxiety disorders typically first emerge and the role of parents is paramount. Using standardised assessments to identify children in the community with elevated anxiety, and those who met diagnostic criteria, we were able to establish barriers experienced by families who do not seek professional help, and those who seek help but do not successfully access it. Moreover, we used findings from underpinning qualitative work to develop the questionnaire instrument to help ensure we captured barriers/facilitators relevant to parents of children with anxiety disorders in the community. Indeed, many of the barriers in this study were endorsed by a larger proportion of parents than observed in other studies [13], indicating that we did capture barriers/facilitators pertinent to parents who have and have not sought professional help.

We acknowledge this study has several limitations. We relied on parent report to collect information on help-seeking and support accessed by families and it is possible this may have resulted in either under- or over-estimating frequencies of help-seeking/support accessed. It is also possible that there was participation bias in the study, both at the school and family level. Participation rates in the initial screening stage of the study were relatively low, with a response rate of 11.5% among schools and 18.2% among families. Schools with an awareness of anxiety difficulties in children may have been more likely to respond to the invitation and participate; and therefore we may not have captured the experiences of families in schools where there is the least support in place to facilitate help-seeking and access to anxiety support. Although we did not target parents of children with anxiety difficulties in the initial screening stage, it is possible that these families were more likely to participate, and the proportion of children screened who met diagnostic criteria and were included in the study was slightly higher than the estimated prevalence rates for child anxiety disorders (7.3% compared to 6.5%) [35]. Parents who had some concerns about their child’s anxiety may also have been more likely to take part in the follow-up study, and as a result we may not have fully represented the experiences of families who have not considered that their child may be experiencing difficulties with anxiety. Indeed, although it was not possible to assess the level of parental concern among those who did not take part in the follow-up, parent-reported child anxiety symptoms (SCAS-P) were significantly lower among those who were invited but did not take part in the follow-up, than those who took part. Also, although schools with a varied demographic profile took part in the study, families with lower socio-economic status and from non-White backgrounds were under-represented within the sample, and therefore our findings may not reflect help-seeking experiences within these groups. Although we worked with individual schools to develop strategies to try to maximise participation (e.g., by distributing study information and questionnaires electronically and as paper copies, researchers visiting the school to talk to children and school staff, offering payments to reimburse schools and participants for their time, sending reminders), we did not have sufficient resource to support further, targeted activities that may have helped improve the representativeness of the sample.

### Implications

The study findings identify a number of key areas to target to promote both help-seeking and access to professional support for child anxiety. In relation to promoting parental help-seeking, this study confirmed the widespread need for readily accessible tools to help parents make judgements about when a child’s anxiety warrants some concern, and may benefit from professional support [18]. Moreover, the findings further illustrate the need to improve public awareness and understanding of how to seek professional support and the type of professional support that is available for child anxiety. In particular, this study highlights the importance of targeting negative attitudes and stigma surrounding seeking support for child anxiety, both in relation to parents seeking support for themselves to enable them to help their child and seeking direct support for children; and the need to be sensitive to the fact that parents without personal experience of using mental health services may be less likely to seek support for their child. This study also illustrates the particular importance of ensuring primary school staff are equipped with the skills and resources to enable them to recognise anxiety difficulties in children, as well as to provide guidance for families on tools, strategies and sources of support available. In relation to improving access to professional support, our findings also illustrate the need for (i) increased provision for child anxiety, (ii) efforts to ensure available support is evidence-based, and (iii) services to offer support for parents to equip them with the skills to support their children. Given that families most commonly seek help and receive support through school, further work is needed to develop and evaluate targeted approaches to identify child anxiety problems and deliver evidence-based treatments within school settings.

## Conclusions

This study provides current data on parental help-seeking for child anxiety in a community sample from across England. Findings identify a substantial unmet need in relation to this common child mental health problem. The majority of children (> 60%) had not received any professional support, only a very small proportion (< 3%) had received evidence-based treatment, and about a third of parents had not sought professional help for their child’s anxiety. Our findings illustrate the range of barriers to both seeking, and accessing support for child anxiety difficulties, and importantly identify keys areas to target to improve access to evidence-based child anxiety treatment. In particular, the findings identify the need for readily available child anxiety identification tools, guidance for families and school staff on the help-seeking process, and increased evidence-based provision that incorporates direct support for parents and is available in school settings.

## Electronic supplementary material

Below is the link to the electronic supplementary material.

Supplementary file1 (DOC 78 kb)

Supplementary file2 (DOC 47 kb)

Supplementary file3 (DOC 52 kb)

Supplementary file4 (DOC 49 kb)

## Footnotes

1 We used published cut-off scores on the SCAS-P and a subset of items on the teacher questionnaire (SCAS-T-8) to identify families to follow-up. Both measures display a good-acceptable level of accuracy in identifying children with anxiety disorders [21].

2 There was no evidence of multicollinearity in the regression model examining the contribution of individual child/parent characteristics in identifying help-seekers/non help-seekers. Tolerance statistics for two barrier items (*It is difficult to get a referral to a specialist service, There are long waiting times for specialist services*) indicated possible multicollinearity in the regression model examining the contribution of individual barriers. This logistic regression was repeated including only one of these items (*It is difficult to get a referral to a specialist service*) and the results were consistent with the original model so only the original regression model is reported here.

## References

[1] Kessler RC, Chiu WT, Demler O, Walters EE (2005). Prevalence, severity and comorbidity of 12 month DSMIV disorders. Arch Gen Psychiatry.

[2] Copeland WE, Angold A, Shanahan L, Costello EJ (2014). Longitudinal patterns of anxiety from childhood to adulthood: the great smoky mountains study. J Am Acad Child Adolesc Psychiatry.

[3] Fineberg NA, Haddad PM, Carpenter L, Gannon B, Sharpe R, Young AH (2013). The size, burden and cost of disorders of the brain in the UK. J Psychopharmacol.

[4] James AC, James G, Cowdrey FA, Soler A, Choke A (2013) Cognitive behavioural therapy for anxiety disorders in children and adolescents. Cochrane Database Syst Rev 6:CD004690. 10.1002/14651858.CD004690.pub3 23733328

[5] Barrett PM, Duffy AL, Dadds MR, Rapee RM (2001). Cognitive-behavioral treatment of anxiety disorders in children: long-term (6-year) follow-up. J Consult Clin Psychol.

[6] Wolk CB, Kendall PC, Beidas RS (2015). Cognitive-behavioral therapy for child anxiety confers long-term protection from suicidality. J Am Acad Child Adolesc Psychiatry.

[7] Green H, McGinnity A, Meltzer H, Ford T, Goodman R (2004) Mental health of children and young people in great Britain. doi:10.1037/e557702010-001

[8] Merikangas KR, He J, Burstein M, Swendsen J, Avenevoli S, Case B (2011). Service utilization for lifetime mental disorders in U.S. adolescents: results of the National Comorbidity Survey-Adolescent Supplement (NCS-A). J Am Acad Child Adolesc Psychiatry.

[9] Lawrence D, Johnson S, Hafekost J, de Haan KB, Sawyer M, Ainley J (2015). The mental health of children and adolescents. Report on the second Australian child and adolescent survey of mental health and wellbeing.

[10] Brown NM, Green JC, Desai MM, Weitzman CC, Rosenthal MS (2014). Need and unmet need for care coordination among children with mental health conditions. Pediatrics.

[11] Chavira DA, Stein MB, Bailey K, Stein MT (2004). Child anxiety in primary care: prevalent but untreated. Depress Anxiety.

[12] Children and Young People’s Mental Health Taskforce (2015) Future in mind: promoting, protecting and improving our children and young people’s mental health and wellbeing

[13] Reardon T, Harvey K, Baranowska M, O’Brien D, Smith L, Creswell C (2017). What do parents perceive are the barriers and facilitators to accessing psychological treatment for mental health problems in children and adolescents? A systematic review of qualitative and quantitative studies. Eur Child Adolesc Psychiatry.

[14] Ryan SM, Jorm AF, Toumbourou JW, Lubman DI (2015). Parent and family factors associated with service use by young people with mental health problems: a systematic review. Early Interv Psychiatry.

[15] Gronholm Petra C., Ford Tamsin, Roberts Ruth E., Thornicroft Graham, Laurens Kristin R., Evans-Lacko Sara (2015). Mental Health Service Use by Young People: The Role of Caregiver Characteristics. PLOS ONE.

[16] Ford T, Hamilton H, Meltzer H, Goodman R (2008). Predictors of service use for mental health problems among British schoolchildren. Child Adolesc Ment Health.

[17] Salloum A, Johnco C, Lewin AB, McBride NM, Storch EA (2016). Barriers to access and participation in community mental health treatment for anxious children. J Affect Disord.

[18] Reardon Tessa, Harvey Kate, Young Bridget, O’Brien Doireann, Creswell Cathy (2018). Barriers and facilitators to parents seeking and accessing professional support for anxiety disorders in children: qualitative interview study. European Child & Adolescent Psychiatry.

[19] Spence SH (1998). A measure of anxiety symptoms among children. Behav Res Ther.

[20] Nauta MH, Scholing A, Rapee RM, Abbott M, Spence SH, Waters A (2004). A parent-report measure of children’s anxiety: psychometric properties and comparison with child-report in a clinic and normal sample. Behav Res Ther.

[21] Reardon T, Spence S, Hesse J, Shakir A, Creswell C (2018). Identifying children with anxiety disorders using brief versions of the Spence Children’s Anxiety Scale for children, parents, and teachers. Psychol Assess.

[22] Arendt K, Hougaard E, Thastum M (2014). Psychometric properties of the child and parent versions of Spence Children’s Anxiety Scale in a Danish community and clinical sample. J Anxiety Disord.

[23] Spence SH, Barrett PM, Turner CM (2003). Psychometric properties of the Spence Children’s Anxiety Scale with young adolescents. J Anxiety Disord.

[24] Whiteside S, Brown A (2008). Exploring the utility of the Spence Children’s Anxiety Scales parent- and child-report forms in a North American sample. J Anxiety Disord.

[25] Silverman WK, Saavedra LM, Pina AA (2001). Test-retest reliability of anxiety symptoms and diagnoses with the Anxiety Disorders Interview Schedule for DSM-IV: child and parent versions. J Am Acad Child Adolesc Psychiatry.

[26] Lyneham HJ, Rapee RM (2005). Agreement between telephone and in-person delivery of a structured interview for anxiety disorders in children. J Am Acad Child Adolesc Psychiatry.

[27] Evans R, Thirlwall K, Cooper P, Creswell C (2017). Using symptom and interference questionnaires to identify recovery among children with anxiety disorders. Psychol Assess.

[28] Langley AK, Falk A, Peris T, Wiley JF, Kendall PC, Ginsburg G (2014). The Child Anxiety Impact Scale: examining parent- and child-reported impairment in child anxiety disorders. J Clin Child Adolesc Psychol.

[29] Langley AK, Bergman RL, McCracken J, Piacentini JC (2004). Impairment in childhood anxiety disorders: preliminary examination of the Child Anxiety Impact Scale-Parent Version. J Child Adolesc Psychopharmacol.

[30] Henry JD, Crawford JR (2005). The short-form version of the Depression Anxiety Stress Scales (DASS-21): construct validity and normative data in a large non-clinical sample. Br J Clin Psychol.

[31] Sinclair SJ, Siefert CJ, Slavin-Mulford JM, Stein MB, Renna M, Blais MA (2012). Psychometric evaluation and normative data for the Depression, Anxiety, and Stress Scales-21 (DASS-21) in a nonclinical sample of U.S. adults. Eval Health Prof.

[32] Hatch SL, Frissa S, Verdecchia M, Stewart R, Fear NT, Reichenberg A (2011). Identifying socio-demographic and socioeconomic determinants of health inequalities in a diverse London community: the South East London Community Health (SELCoH) study. BMC Public Health.

[33] Ford T, MacDIarmid F, Russell AE, Racey D, Goodman R (2017). The predictors of persistent DSM-IV disorders in 3-year follow-ups of the British Child and Adolescent Mental Health Surveys 1999 and 2004. Psychol Med.

[34] Costello EJ, Maughan B (2015). Annual research review: optimal outcomes of child and adolescent mental illness. J Child Psychol Psychiatry.

[35] Polanczyk GV, Salum GA, Sugaya LS, Caye A, Rohde LA (2015). Annual research review: a meta-analysis of the worldwide prevalence of mental disorders in children and adolescents. J Child Psychol Psychiatry.

