# Supplementary material for: Seeking and accessing professional support for child anxiety in a community sample
Source: Eur Child Adolesc Psychiatry. 2019 Aug 13;29(5):649–64. doi: 10.1007/s00787-019-01388-4 (PMC7250799; doi:10.1007/s00787-019-01388-4)
Supplement: Supplementary file 1 — Supplementary file1 (DOC 78 kb) [file 787_2019_1388_MOESM1_ESM.doc]

Online Resource 1

Factors associated with parental help seeking: bivariate analyses

|  | Anxiety disorder sample (*n*=138) | | | Total sample (*n*=222) | | |
| --- | --- | --- | --- | --- | --- | --- |
|  | Help-seekers  (*n*=89) | Non-help seekers (*n*=49) | Group difference | Help-seekers  (*n*=117) | Non-help seekers (*n*=105) | Group difference |
| Child gender  Female, *n* (%) | 40 (44.9) | 23 (46.9) | *X^2^ = 0*.05, *p = 0*.82 | 50 (42.7) | 57 (54.3) | *X^2^* = 2.96, *p* = 0.09 |
| Child age  mean (SD) | 9.86 (1.15) | 9.66 (1.23) | *t*(134) = 0.97, *p* = 0.33 | 9.75 (1.19) | 9.48 (1.23) | *X^2^* =1.63, *p* = 0.11 |
| Family SES  higher / professional, *n* (%) | 41 (47.1) | 23 (47.9) | *X^2^* = 0.01, *p* = 0.93 | 56 (49.1) | 49 (48.0) | *X^2^* = 0.03, *p* = 0.87 |
| Parent education  Higher education, *n* (%) | 31 (34.8) | 22 (44.9) | *X^2^* = 1.72, *p* = 0.19 | 49 (41.9) | 50 (47.1) | *X^2^* = 0.88, *p* = .35 |
| SCAS-P (total score)  Mean (SD) | 41.89 (17.01) | 34.80 (11.92) | ***t*(136) = 2.59, *p* = 0.01, *d* = 0.48** | 38.64 (16.48) | 28.80 (13.10) | ***t*(220) = 4.89, *p* < 0.001, *d* = 0.66** |
| SCAS-C-27 (total score)  Mean (SD) | 31.05 (15.20) | 26.38 (13.53) | *t*(114) = 1.66, *p* = 0.10 | 30.02 (14.70) | 26.57 (13.48) | *t*(186) = 1.66, *p* = 0.10 |
| SCAS-T-20 (total score)  Mean (SD) | 12.97 (8.68) | 10.18 (8.41) | *t*(115) = 1.66, *p* = 0.10 | 13.46 (8.56) | 10.18 (7.03) | ***t*(196) = 2.92, *p* = 0.004, *d* = 0.42** |
| CAIS-P  Total score, Mean (SD)  School, Mean (SD)  Social, Mean (SD)  Home/family, Mean (SD) | 28.25 (14.25)  12.51 (7.08)  8.22 (6.04)  7.47 (4.38) | 22.73 (11.47)  10.63 (6.07)  7.46 (4.50)  4.57 (3.87) | ***t*(134) = 2.32, *p* = 0.02, *d* = 0.43**  *t*(130) = 1.53, *p* = 0.13  *t*(132) = 0.76, *p* = 0.45  ***t*(136) = 3.88, *p* < 0.001, *d* = 0.70** | 26.20 (14.76)  12.04 (7.23)  7.54 (6.18)  6.59 (4.46) | 16.28 (11.62)  7.83 (6.26)  5.07 (4.01)  3.34 (3.47) | ***t*(218) = 5.51, *p* < 0.001, *d* = 0.75**  ***t*(213) = 4.53, *p* < 0.001, *d* = 0.62**  ***t*(215) = 3.35, *p* = 0.001, *d* = 0.47**  ***t*(215) = 6.00, *p* < 0.001, *d* = 0.81** |
| Perceived need for professional help (child)  Mean (SD) | 3.25 (1.16) | 2.55 (1.02) | ***t*(136) = 3.51, *p* = 0.001, *d* = 0.64** | 3.17 (1.14) | 2.23 (1.18) | ***t*(220) = 6.05, *p* < 0.001, *d* = 0.81** |
| Perceived need for professional help (parent)  Mean (SD) | 3.16 (1.12) | 2.73 (1.08) | ***t*(136) = 2.16, *p* = 0.03, *d* = 0.39** | 3.08 (1.11) | 2.46 (1.23) | ***t*(219) = 3.96, *p* < 0.001, *d* = 0.53** |
| DASS-21  Total score, Mean (SD)  Anxiety, Mean (SD)  Stress, Mean (SD)  Depression, Mean (SD) | 17.16 (13.41)  3.60 (4.15)  8.40 (5.64)  5.17 (5.27) | 12.81 (9.75)  2.82 (2.95)  6.88 (4.65)  3.06 (3.47) | ***t*(134) = 1.99, *p* = 0.05, *d* = 0.37**  *t*(134) = 1.16, *p* = 0.25  *t*(134) = 1.61, *p* = 0.11  ***t*(133) = 2.49, *p* = 0.01, *d* = 0.47** | 15.63 (12.62)  3.04 (3.83)  7.91 (5.53)  4.68 (4.97) | 11.99 (10.29)  2.46 (3.07)  6.26 (4.66)  3.24 (3.89) | ***t*(217) = 2.32, *p* = 0.02, *d* = 0.32**  *t*(217) = 1.23, *p* = 0.22  ***t*(217) = 2.38, *p* = 0.02, *d* = 0.32**  ***t*(216) = 2.36, *p* = 0.02, *d* = 0.32** |
| Parent contact with professional about mental health problems, *n*(%)  GP *n* (%)  MH Specialist, *n* (%) | 66 (75.0)  61 (68.6)  52 (58.4) | 28 (57.1)  25 (51.0)  16 (32.7) | ***X^2^* = 4.04, *p* = 0.04**  ***X^2^* = 3.97, *p* = 0.05**  ***X^2^* = 8.24, *p* = 0.004** | 83 (70.9)  75 (64.1)  65 (55.6) | 64 (61.0)  52 (49.5)  35 (33.3) | *X^2^* = 2.19, *p* = 0.14  ***X^2^* = 4.50, *p* = 0.03**  ***X^2^* = 10.70, *p* = 0.001** |
| Parent rated professional support not helpful^a^, *n* (%)  Parent rated professional support very helpful/ extremely helpful^b^,  *n* (%) | 7 (7.9)  29 (32.6) | 3 (6.1)  11 (22.4) | *X^2^* = 0.14, *p* = 0.71  *X^2^* = 0.58, *p* = 0.21 | 7 (6.0)  42 (35.9) | 5 (4.8)  31 (29.5) | *X^2^* = 0.16, *p* = 0.69  *X^2^* = 1.02, *p* = 0.31 |
| Total barriers | 47.02 (25.66) | 36.43 (22.10) | ***t*(132) =2.38, *p* = 0.02, *d* = 0.44** | 43.74 (25.55) | 29.24 (22.42) | ***t*(216) = 4.28, *p* < 0.001, *d* = 0.60** |
| Recognising child’s anxiety difficulties barriers | 8.31 (5.47) | 8.39 (4.61) | *t*(131) = 0.09, *p* = 0.93 | 8.04 (5.43) | 6.70 (4.88) | *t*(213) = 1.88, *p* = 0.06 |
| Recognising the need for professional support barriers | 12.74 (5.58) | 11.96 (7.97) | *t*(131) = 0.51, *p* = 0.61 | 11.85 (8.38) | 9.80 (7.82) | *t*(214) = 1.85, *p* = 0.07 |
| Contacting professionals barriers | 12.11 (8.04) | 8.93 (6.94) | ***t*(129) = 2.24, *p* = 0.03, *d* = 0.42** | 10.95 (7.97) | 6.66 (6.62) | ***t*(213) = 4.25, *p* < 0.001, *d* = 0.59** |
| Receiving support barriers | 14.55 (9.09) | 7.66 (8.00) | ***t*(132) = 4.26, *p* < 0.001, *d* = 0.80** | 13.43 (8.84) | 6.32 (7.61) | ***t*(208) = 6.20, *p* < 0.001, *d* = 0.86** |

*Note.* SES=socio-economic status; MH Specialist = mental health specialist

*^a^* Comparison group = Parents who had not received professional support themselves + those who had received support and rated it as slightly helpful/moderately helpful/very helpful/extremely helpful. ^b^ Comparison group = Parents who had not received professional support themselves+those who had received support and rated it as not helpful/slightly helpful/moderately helpful
