# Supplementary material for: Seeking and accessing professional support for child anxiety in a community sample
Source: Eur Child Adolesc Psychiatry. 2019 Aug 13;29(5):649–64. doi: 10.1007/s00787-019-01388-4 (PMC7250799; doi:10.1007/s00787-019-01388-4)
Supplement: Supplementary file 2 — Supplementary file2 (DOC 47 kb) [file 787_2019_1388_MOESM2_ESM.doc]

Online Resource 2

Logistic regression examining contribution of child and parent characteristics in identifying help-seekers and non-help seekers (total sample)

|  | *b* (Wald statistic) | Adjusted Odds Ratio  (95% CI) | R^2^ | Model |
| --- | --- | --- | --- | --- |
|  |  |  |  |  |
| SCAS-P | 0.18 (1.69), *p* = 0.19 | 1.01 (0.99-1.05) | 0.30 (Cox & Snell)  0.39 (Nagelkerke) | *X^2^*(7) =  75.29 |
| CAIS-P-Home/family | **0.14 (6.74), *p* = 0.009** | **1.14 (1.03-1.27)** |
| DASS-21-Total | 0.001 (0.003), *p* = 0.96 | 1.00 (0.97-1.03) |
| Parent contact with mental health specialist | **0.97 (7.77), *p* = 0.005** | **2.63 (1.33-5.19)** |  |  |
| Perceived need for professional help (child) | **1.44 (15.76), *p* < 0.001** | **4.23 (2.08-8.63)** |  |  |
| Perceived need for professional help (parent) | **-1.04 (7.90), *p* = 0.005** | **0.35 (0.17-0.73)** |  |  |
| Anxiety diagnosis | 0.46 (1.56), *p* = 0.21 | 1.59 (0.77-3.29) |
