# Supplementary material for: Seeking and accessing professional support for child anxiety in a community sample
Source: Eur Child Adolesc Psychiatry. 2019 Aug 13;29(5):649–64. doi: 10.1007/s00787-019-01388-4 (PMC7250799; doi:10.1007/s00787-019-01388-4)
Supplement: Supplementary file 3 — Supplementary file3 (DOC 52 kb) [file 787_2019_1388_MOESM3_ESM.doc]

Online Resource 3

Factors associated with parent reported barriers (total barrier scores): bivariate analyses

|  | Anxiety disorder sample  (*n*=138) | Total sample  (*n*=222) |
| --- | --- | --- |
| Child gender  Female, Mean (SD)  Male, Mean (SD) | 43.20 (26.06)  43.45 (24.13)  *t*(132) = 0.08, *p* = 0.94 | 35.30 (25.34)  38.49 (24.99)  *t*(216) = 0.93, *p* = 0.35 |
| Child age | *r* = -0.04, *p* = 0.62 | *r* = 0-.00, *p* = 0.93 |
| Family SES  higher / professional, Mean (SD)  other, Mean (SD) | 40.38 (22.88)  43.91 (24.81)  *t*(129) = 0.85, *p* = 0.40 | 38.44 (25.05)  34.23 (23.48)  *t*(210) = 1.26, *p* = 0.21 |
| Parent education  Higher education, Mean (SD)  School/further education, Mean (SD) | 36.44 (19.69)  46.83 (26.63)  ***t*(129) = 2.41, *p* = 0.02** | 31.86 (21.07)  40.29 (27.06)  ***t*(213) = 2.51, *p* = 0.01** |
| SCAS-P (total score) | ***r* = 0.21, *p* = 0.02** | ***r* = 0.36, *p* < 0.001** |
| SCAS-C-27 (total score) | ***r* = 0.19, *p* = 0.05** | ***r* = 0.20, *p* = 0.007** |
| SCAS-T-20 (total score) | *r* = 0.10, *p* = 0.30 | *r* = 0.02, *p* = 0.78 |
| CAIS-P  Total score  School  Social  Home/family | ***r* = 0.32, *p* < 0.001**  ***r* = 0.31, *p* < 0.001**  ***r* = 0.18*, p* = 0.04**  ***r* = 0.30, *p* < 0.001** | ***r* = 0.47, *p* < 0.001**  ***r* = 0.45, *p* < 0.001**  ***r* = 0.33*, p* < 0.001**  ***r* = 0.40, *p* < 0.001** |
| Perceived need for professional help (child) | *r* = 0.14, *p* = 0.10 | ***r* = 0.27, *p* < 0.001** |
| Perceived need for professional help (parent) | ***r* = 0.18, *p* = 0.04** | ***r* = 0.29, *p* < 0.001** |
| DASS-21 (total score) | ***r* = 0.49, *p* <0.001** | ***r* = 0.40, *p* < 0.001** |
| Parent contact with mental health specialist  No contact  Contact | 37.58 (23.40)  48.79 (25.16)  ***t*(130) = 2.65, *p* = 0.009** | 31.89 (23.51)  42.92 (25.61)  ***t*(213) = 3.29, *p* = 0.01** |

*Note.* SES=socio-economic status
