# Supplementary material for: Seeking and accessing professional support for child anxiety in a community sample
Source: Eur Child Adolesc Psychiatry. 2019 Aug 13;29(5):649–64. doi: 10.1007/s00787-019-01388-4 (PMC7250799; doi:10.1007/s00787-019-01388-4)
Supplement: Supplementary file 4 — Supplementary file4 (DOC 49 kb) [file 787_2019_1388_MOESM4_ESM.doc]

Online Resource 4

Individual barriers associated with parental help seeking (total sample, *n*=222)

|  | *b* (Wald statistic) | Adjusted Odds Ratio  (95% CI) | R^2^ | Model |
| --- | --- | --- | --- | --- |
| Parent education | 0.14 (0.14), *p* = 0.70 | 1.15 (0.56-2.35) | 0.30 (Cox & Snell)  0.40 (Nagelkerke) | *X^2^*(11) =  70.62 |
| **Anxiety diagnosis** | **1.14 (9.56), *p* = 0.02** | **3.12 (1.52-6.43)** |  |  |
| My child’s anxiety comes and goes in phases | 0.23 (1.27), *p* = 0.26 | 1.25 (0.85-1.85) |  |  |
| I’m not sure if my child’s anxiety is normal | 0.27 (1.72), *p* = 0.19 | 1.31 (0.88-1.94) |  |  |
| I don’t want my child to think she/he has a problem | 0.04 (0.05), *p* = 0.83 | 1.04 (0.72-1.52) |  |  |
| **My child’s anxiety may improve without professional help** | **-0.52 (6.45), *p* = 0.01** | **0.60 (0.40-0.89)** |  |  |
| **Teachers or other professionals have never suggested my child would benefit from professional help** | **-0.56 (9.32), *p*= 0.02** | **0.57 (0.40-0.82)** |  |  |
| I don’t know who to ask for help | -0.29 (1.15), *p* = 0.28 | 0.75 (0.44-1.27) |  |  |
| I don’t know what help is available for children with anxiety difficulties | 0.25 (0.83), *p* = 0.36 | 1.29 (0.75-2.23) |  |  |
| **It is difficult to get a referral to a specialist service** | **0.86 (7.58), *p* = 0.06** | **2.36 (1.28-4.35)** |  |  |
| There are long waiting times for specialist services | 0.14 (0.24), *p* = 0.63 | 1.14 (0.67-1.97) |  |  |
